# Hedgehog signalling mediates drug resistance through targeting TAP1 in hepatocellular carcinoma

**DOI:** 10.1111/jcmm.15090

**Published:** 2020-02-28

**Authors:** Xiao‐Tian Zhou, Jia Ding, Hui‐Yan Li, Jie‐Liang Zuo, Sheng‐Yang Ge, Hu‐Liang Jia, Jian Wu

**Affiliations:** ^1^ MOE/NHC/CAMS Key Laboratory of Medical Molecular Virology Department of Medical Microbiology and Parasitology School of Basic Medical Sciences Shanghai Medical College Fudan University Shanghai China; ^2^ Department of Gastroenterology Shanghai Jing'an District Central Hospital Fudan University Shanghai China; ^3^ Department of General Surgery Huashan Hospital of Fudan University Shanghai China; ^4^ Department of Gastroenterology & Hepatology Zhongshan Hospital of Fudan University Shanghai China; ^5^ Shanghai Institute of Liver Diseases Shanghai Medical College Fudan University Shanghai China

**Keywords:** chemo‐resistance, GANT61, hedgehog signalling, hepatocellular carcinoma, TAP1

## Abstract

Multidrug resistance is one of the reasons for low survival of advanced hepatocellular carcinoma (HCC). Our previous studies indicate that the hedgehog signalling is involved in hepatic carcinogenesis, metastasis and chemo‐resistance. The present study aims to uncover molecular mechanisms underlying hepatoma chemo‐resistance. TAP1 and GLI1/2 gene expression was assessed in both poorly differentiated hepatoma cells and HCC specimens. Potential GLI‐binding site in the TAP1 promoter sequence was validated by molecular assays. Approximately 75% HCC specimens exhibited an elevated expression of hedgehog GLI1 transcription factor compared with adjacent liver tissue. Both GLI1/2 and TAP1 protein levels were significantly elevated in poorly differentiated hepatoma cells. Both Huh‐7‐trans and Huh‐7‐DN displayed more karyotypic abnormalities and differential gene expression profiles than their native Huh‐7 cells. Sensitivity to Sorafenib, doxorubicin and cisplatin was remarkably improved after either GLI1 or TAP1 gene was inhibited by an RNAi approach or by a specific GLI1/2 inhibitor, GANT61. Further experiments confirmed that hedgehog transcription factor GLI1/2 binds to the TAP1 promoter, indicating that TAP1 is one of GLI1/2 target genes. In conclusion, TAP1 is under direct transcriptional control of the hedgehog signalling. Targeting hedgehog signalling confers a novel insight into alleviating drug resistance in the treatment of refractory HCC.

## 1 INTRODUCTION

With an increasing incidence, hepatocellular carcinoma (HCC) ranks the third cancer‐related death.^1^ Majority of patients were inoperable at the time of diagnosis, and only 30% of them can benefit from established curable treatments, such as surgical resection and liver transplantation. For patients at an intermediate stage of HCC, various choices of adjuvant therapies including percutaneous ethanol injection, radiofrequency ablation, cryoablation, transarterial chemoembolization (TACE) or selective internal radiation therapy (SIRT) are available. However, none of these adjuvant therapies has significantly improved an overall 5‐year survival rate in patients with advanced HCC. Sorafenib, a small inhibitor of multi‐tyrosine kinases approved by the US Food and Drug Administration (FDA), did extend 3 months of survival in patients with advanced HCC. Nevertheless, shrinkage in tumour size was actually demonstrated only in 1% patients. Moreover, adverse events and resistance to Sorafenib developed in most patients with progressive deterioration.

A previous study demonstrated that hedgehog (Hh) signalling components were detected in 50%‐70% of specimens in advanced HCC, indicating that Hh signalling activation in HCC is not uncommon.^2^ Our previous studies implicated Hh signalling plays an important role in enhancing hepatic carcinogenesis, epithelial‐mesenchymal transition (EMT) and invasion.^3^ However, available studies could not provide a clear picture of Hh molecular interplays in modulating HCC drug resistance. In our previous studies, two poorly differentiated hepatoma subpopulations, Huh‐7‐trans and Huh‐7‐DN with aberrant Hh activation, were isolated from their parental Huh‐7 cells by fluorescence‐activated cell sorting (FACS) with a negative CD133/EpCAM surface marker profile. These two subpopulations displayed enhanced chemo‐resistance and invasive behaviour compared with their counterparts.^4^, ^5^ Our further study demonstrated that Hh signalling transcription factor GLI2 modulates drug resistance in Huh‐7‐DN cells through ABCC1 (ATP‐binding cassette, ABC subfamily C member 1).^5^ Therefore, it is intriguing to explore whether the Hh signalling controls drug resistance in poorly differentiated hepatoma cells with a negative CD133/EpCAM surface marker profile through a particular molecular mechanism. Our working hypothesis lies in that subpopulations derived of heterogeneous hepatoma mass may possess primary resistance to chemotherapies under the hedgehog signalling control. Resistant subpopulations which are often poorly differentiated tend to escape from chemotherapies and overgrow due to a forced selection in exposure to chemotherapy.^6^

Our preliminary RNA‐sequencing (RNA‐Seq) study revealed that another ABC member, TAP1 (ABCB2, ABC subfamily B member) highly expressed in both Huh‐7‐trans and Huh‐7‐DN subpopulations. Therefore, the aim of the present study was to elucidate the underlying mechanism of the hedgehog signalling in governing chemosensitivity through modulating TAP1 expression in both Huh‐7‐trans and Huh‐7‐DN subpopulations, the representatives of poorly differentiated hepatoma cells. To test our hypothesis, karyotypic analysis, RNA‐seq analysis, drug sensitivity assay, GLI1 and TAP1 silencing by lentiviral transduction were employed to explore crucial role of the hedgehog signalling in mediating chemo‐resistance. The findings of this study deepen our understanding of the drug resistance modulation by the hedgehog signalling and provide a promising strategy for improving chemosensitivity of HCC.

## 2 MATERIALS AND METHODS

### 2.1 Cell culture

Hep3B hepatoma cell line was obtained from ATCC, and Huh‐7 hepatoma cell line was a gift from Prof. Mark Feitelson (Temple University, PA, USA). HLE and HLF hepatoma cell lines were obtained from the Japanese Collection of Research Bioresource bank. The above‐mentioned cell lines were all authenticated using Short Tandem Repeat (STR) analysis (GENEWIZ, Inc) and tested periodically for mycoplasma by polymerase chain reaction (PCR). Huh‐7‐DN subpopulation was enriched with EpCAM^−^/CD133^−^ surface markers from Huh‐7 cells by FACS, while Huh‐7‐trans cells were further selected using the transwell migration assay after FACS enrichment as we previously reported.^4^, ^5^

### 2.2 Human samples

A total of 12 pairs of HCC specimens and matched pericancerous tissues were obtained from the tissue repository of the Department of Hepatobiliary Surgery, Huashan Hospital of Fudan University, Shanghai, China. All specimens were cut into pieces of 3 mm in diameter for RNA and protein extraction, or fixed in 4% paraformaldehyde for immunohistochemistry. Corresponding clinical data and pathologic diagnosis were obtained from medical record with written consent prior to surgical procedures. The human subject protocol (2018‐M01) was approved by the Ethic Committee of Fudan University School of Basic Medical Sciences.

### 2.3 Karyotype analysis

After pre‐treated with colchicine (0.2 μg/mL) for 3 hours, Huh‐7, Huh‐7‐trans and Huh‐7‐DN cells were digested and incubated in 75 mmol/L hypotonic KCL solution at 37°C for 15 minutes. Then, cells were fixed in methanol/acetic acid (1:3 in volume) in 30 minutes for 3 times. The chromosomal abnormality of fixed cells was analysed by karyotyping (ADICON Clinical Laboratories Inc) according to the reference of International System for Human Cytogenetic Nomenclature (ISCN) 2013.

### 2.4 RNA‐Seq analysis

To find potential genes involved in drug resistance of Huh‐7‐trans and Huh‐7‐DN cells, total mRNA of Huh‐7, Huh‐7‐trans and Huh‐7‐DN cells was extracted by Trizol^®^ Reagent (Invitrogen) according to the instruction manual. After verification of integrity and purity, total mRNA was randomly fractured into fragments of approximately 200 bp and transcribed to double‐strand DNA with added adaptors. Sequences of resulting DNA products were probed through Illumina Hiseq4000 by Majorbio Ltd. Differential gene expression was analysed through edgeR software using gene read count. False discovery rate (FDR) < 0.01 & log_2_|FC| ≥ 2 was considered as significantly differential gene expression. Gene ontology (GO) functional analysis was completed by Fisher's exact test using GOatools Software, and *P* value was adjusted by four tests (Bonferroni, Holm, Sidak and false discovery rate) to control false positive rate, when corrected *P* value (p_fdr) ≤0.05 was considered to be significant for GO enrichment. Kyoto Encyclopaedia of Genes and Genomes (KEGG) pathway was analysed by Fisher's exact test using KOBAS software. The corrected *P* ≤ .05 was considered to be significant for KEGG enrichment.

### 2.5 Quantitative reverse transcription polymerase chain reaction (qRT‐PCR)

Total RNA of hepatoma cells was isolated using RNA Prep Pure Cell kit (TIANGEN), and total RNA of human HCC tissues was isolated by the TRIzol reagent (Thermo‐Fisher) according to the manufacturer's instruction. Then, total RNA was transcribed to cDNA by PrimeScript RT reagent kit (TAKARA). Quantitative RT‐PCR was performed using the Power SYBR Green Master Mix (Invitrogen Inc) in the Eppendorf AG 22331 RT‐PCR system. Relative mRNA expression of ABC transporters, hedgehog signalling molecules, epithelial‐mesenchymal transition (EMT) and hepatocyte‐specific proteins was normalized to glyceraldehyde‐3‐phosphate dehydrogenase (GAPDH) as a house‐keeping gene control and calculated using 2^(−ΔΔCT)^ methods as previously described.^4^ All primer pair sequences were listed in Table S1.

### 2.6 Immunohistochemical staining

Hepatocellular carcinoma specimens and paired pericancerous tissue were fixed in 4% formalin, sectioned and embedded in paraffin for immunohistochemistry (IHC). Sections were deparafinized in xylene and rehydrated by ethanol at different concentration. Slides were immersed in 3% H_2_O_2_ to block endogenous peroxidase activity, followed by heating sodium citrate buffer at pH 6.0 with 0.05% Tween 20 for antigen retrieval. Non‐specific protein binding was blocked by 3% bovine serum albumin (BSA), and sections were incubated at 4°C with primary TAP1 antibody. After overnight incubation, sections were washed three times for 5 minutes each with 1X TBST and further incubated at room temperature with HRP‐conjugated anti‐rabbit IgG antibody for 1 hour. The sections were visualized with colour development by DAB substrate (ZSGB‐Bio) and counterstained with haematoxylin. Images were taken by inverted microscope (Nikon).

### 2.7 Western blot analysis

Total protein of hepatoma cells was extracted by RIPA buffer as we previously described,^7^ and total protein of human HCC specimens was extracted using Minute™ total protein extraction kit (Invent Biotechnologies) according to the manufacturer's protocol. Equal amount of protein (30 μg) was loaded and separated by a 4%‐12% gradient Bis‐Tris gel (Tanon Science & Technology Co., Ltd.), then transferred to a polyvinylidene fluoride (PVDF) membrane. After being blocked by 5% non‐fat milk and incubated with primary antibodies against TAP1, GLI1/2, GAPDH or β‐actin overnight at 4°C, blotted membranes were incubated with horseradish peroxidase (HRP)‐conjugated anti‐mouse or rabbit IgG antibody for 1 hour at room temperature. Protein bands were imaged by High‐sig Enhanced chemiluminescent system (Tanon 5200) as previously reported.^5^ The primary antibodies used were listed in Table S2.

### 2.8 shRNA‐mediated gene knock‐down through lentiviral transduction

Sequences of shRNAs against human GLI1, TAP1 and scramble shRNA were obtained from Sigma‐Aldrich and cloned into pLKO.1 vector. For generation of lentiviruses, the shRNA plasmids and packaging plasmids (pMD2.G and psPAX2) were cotransfected into human embryonic kidney (HEK) 293T cells as we previously reported.^7^ Huh‐7‐DN and Huh‐7‐trans hepatoma cells were transduced with lentivirus harbouring specific shRNA. Fourteen days after puromycin selection, stable clones of Huh‐7‐DN and Huh‐7‐trans cells with lentiviral transduction were collected for determination of GLI1 and TAP1 expression by qRT‐PCR and Western blot analysis. The shRNA sequences were listed in Table S3.

### 2.9 Construction of a reporter system and dual‐luciferase assay

A potential GLI‐binding site on the TAP1 promoter (5′‐TAGACGACCCCCCTCAGAAA‐3′, NG_011759.1:4498‐4517) was identified using the program MatInspector. Two fragments (TAP1_a and TAP1_b) containing the potential GLI‐binding sequence in the TAP1 promoter, and one fragment (TAP1_c) without the binding site were PCR‐amplified and cloned into pGL4.23 vector (Promega). Predicted binding sequence and primers for three TAP1 promoter constructs were listed in Table S4. Three TAP1 promoter constructs were cotransfected with pRL‐TK *renilla* vector into HLE cells by Lipofectamine 3000 (Invitrogen). Forty‐eight hours after transfection, promoter activity was determined by a dual‐luciferase reporter assay system (Promega) as previously described,^5^ where the *renilla* luciferase was used to normalize the transfection efficiency.

### 2.10 Chromatin immunoprecipitation (ChIP) assay

The GLI‐binding site of the TAP1 promoter was verified by a ChIP assay using Epiquik chromatin immunoprecipitation kit (Epigentek). In brief, DNA was sheared into fragments of 250‐1000 bp by a sonicator (SCIENTZ) after cells were collected, cross‐linked and lysed. Monoclonal GLI1 antibody and polyclonal GLI2 antibody were used for immunoprecipitation. Primer sequences used to PCR‐amplify DNA in the precipitated protein were sense 5′‐TGTGATGAGTTGGT‐3′ and anti‐sense 5′‐CGGAGAAGTGAATG‐3′ and resulted in a product size of 368 bp.

### 2.11 Statistical analysis

All the experiments were repeated at least three times independently, and data of continuous variables were represented as mean ± SD. The difference between two groups was measured using a two‐sided *t* test or one‐way ANOVA when analysing data is in more than two groups, and LSD or Bonferroni tests were further employed for multiple comparisons in given two groups. If the assumptions of the normal distribution were violated, Wilcoxon rank‐sum test was used to analyze the difference. All tests were performed by IBM SPSS Statistics 20 and Graphpad Prism 6, and *P* ≤ .05 was considered to be statistically significant.

## 3 RESULTS

### 3.1 Karyotypic features of Huh‐7‐trans and Huh‐7‐DN subpopulations

**Figure 1:**
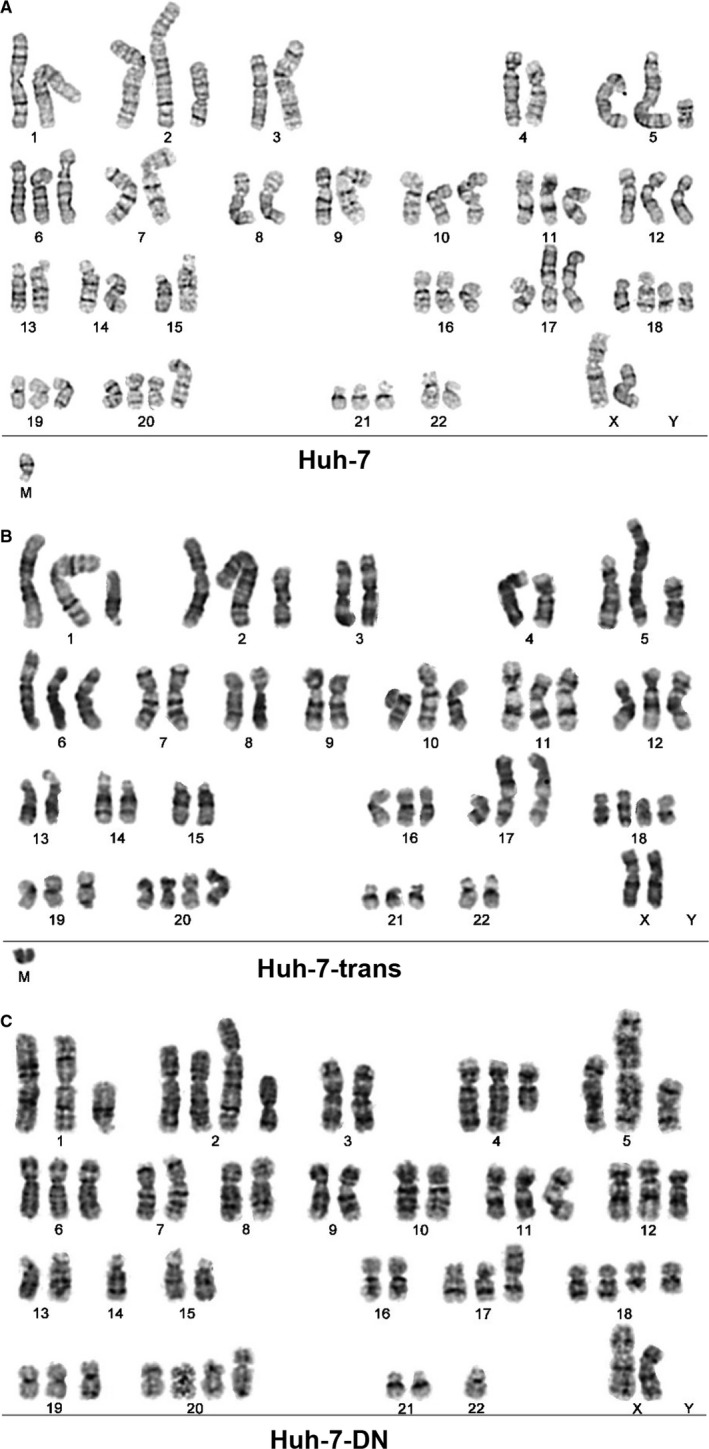
Karyotypic features of Huh‐7, Huh‐7‐trans and Huh‐7‐DN cells. A, Parental Huh‐7 cells (n = 10) were shown to have a variety of abnormalities in karyotypic analysis, such as haploidy, hyperdiploidy, translocation, derivation and deletion. Haploidy (X, 1, 3, 4, 7, 8, 9, 13, 14, 15, 18 and 22), hyperdiploidy 20 and translocation: T(1;2), T(2;7), T(4), T(17), derivation: der (2), der (5), der (17), der (19), der (20), deletions (del 18) and isochromic chromosome 5 and 19 are seen. B, Huh‐7‐trans cells (n = 20) were revealed to have a high frequency of triploid. haploidy (X, 3, 4, 7, 8, 9, 13, 14, 15, 17, 22) was seen. C, Abnormal cytogenetic characteristics were identified in Huh‐7‐DN cells (n = 8)

Huh‐7‐trans and Huh‐7‐DN subpopulations displayed different karyotypes compared with their parental Huh‐7 cells. A variety of karyotypic abnormalities in Huh‐7 cells were observed, such as haploidy, hyperdiploidy, translocation, derivation and isochromic chromosome (5&19) (Figure 1A). However, a high frequency of triploid was noted in Huh‐7‐trans cells, and haploidy, hyperdiploidy 20 (4/20) and der (17) were identified (Figure 1B). Haploidy and der (2), (4), (17), (19), (20), translocation T (1; 2), q arm deletion in chromosome 18 were found in Huh‐7‐DN cells (Figure 1C). The distinct karyotypic abnormalities in Huh‐7‐trans and Huh‐7‐DN are thought to be the genetic signatures for vast heterogeneity between these subpopulations.

### 3.2 Differential gene expression profiles between Huh‐7‐trans, Huh‐7‐DN and Huh‐7 cells

**Figure 2:**
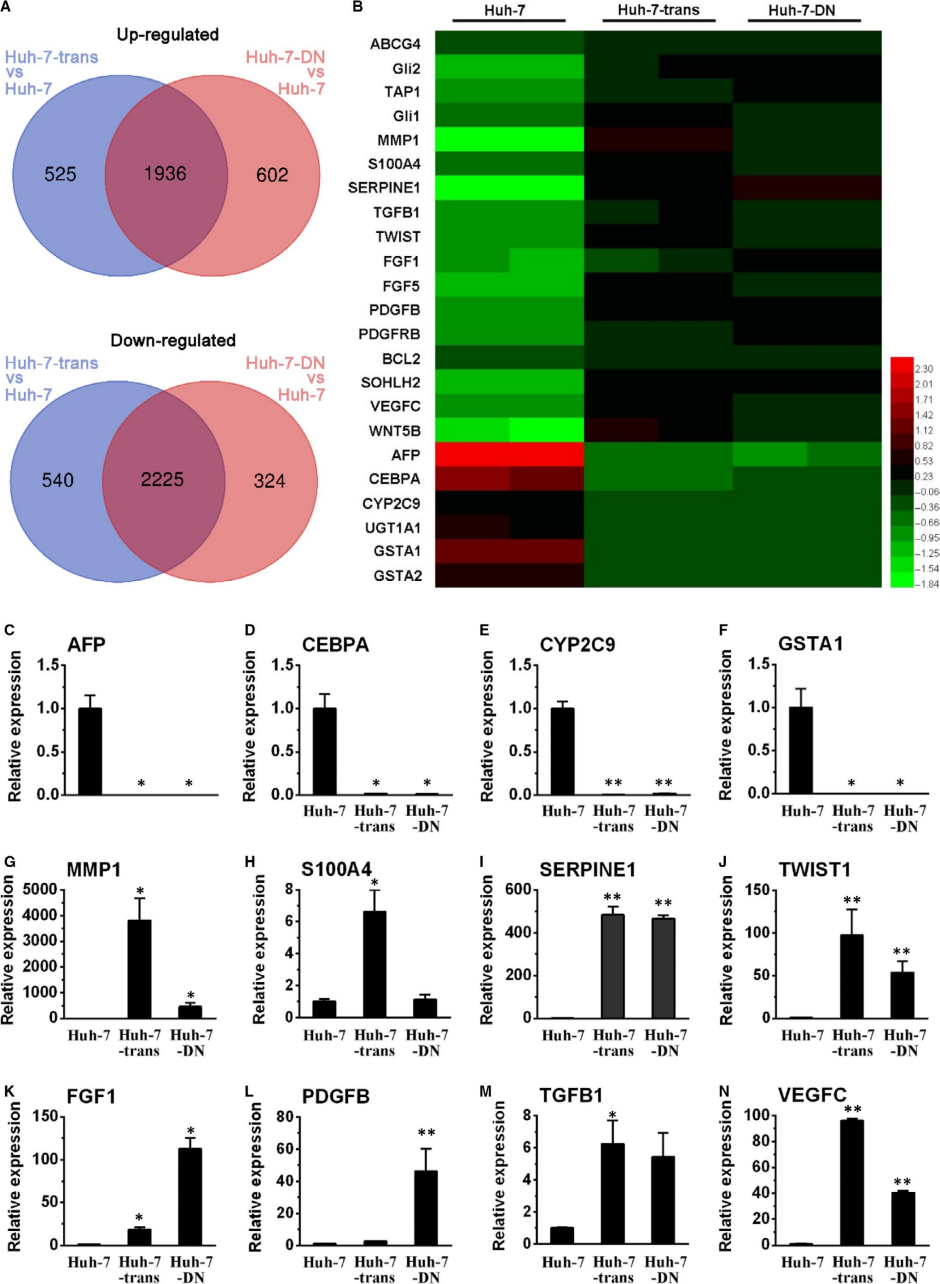
Transcriptome heat map of Huh‐7‐trans and Huh‐7‐DN populations. Two aliquots of Huh‐7, Huh‐7‐trans and Huh‐7‐DN cells were used for RNA extraction in each cell type. B, Heat map of representative differential expression of genes in drug resistance, hedgehog signalling, stemness, cancer progression and hepatocyte‐specific genes from RNA‐sequencing data. A, Venn diagrams of significant differentially expressed genes between Huh‐7‐trans and Huh‐7‐DN vs Huh‐7 cells. FDR < 0.01 & log2|FC| ≥ 2 was considered as significantly differential gene expression. Representative of differential gene expression levels of hepatocyte‐specific genes (C‐F), EMT‐associated transcription factors (G‐J) and cancer progressive genes (K‐N) was verified by quantitative RT‐PCR using Huh‐7 cells as controls (n = 3). *P < .05; **P < .01 compared with controls

To clarify the correlation between the karyotypic abnormality and gene expression profile, RNA‐seq and KEGG pathway analysis were used to explore transcriptome of Huh‐7‐trans and Huh‐7‐DN cells. The similarity in gene expression between Huh‐7‐trans or Huh‐7‐DN cells and parental Huh‐7 cells was 91.4% and 91.6% (Figure 2A). The majority of differentially expressed genes belong to the categories of hepatocyte‐specific genes (AFP, CEBPA, CYP2C9, UGT1A1, GSTA1/2), EMT (MMP1, S100A4, SERPINE1, TWIST1), ABC transporter members (TAP1, ABCG4), hedgehog pathway (GLI1, GLI2), Wnt signalling pathway (WNT5B) and stemness (SOHLH2) (Figure 2B).

**Figure 3:**
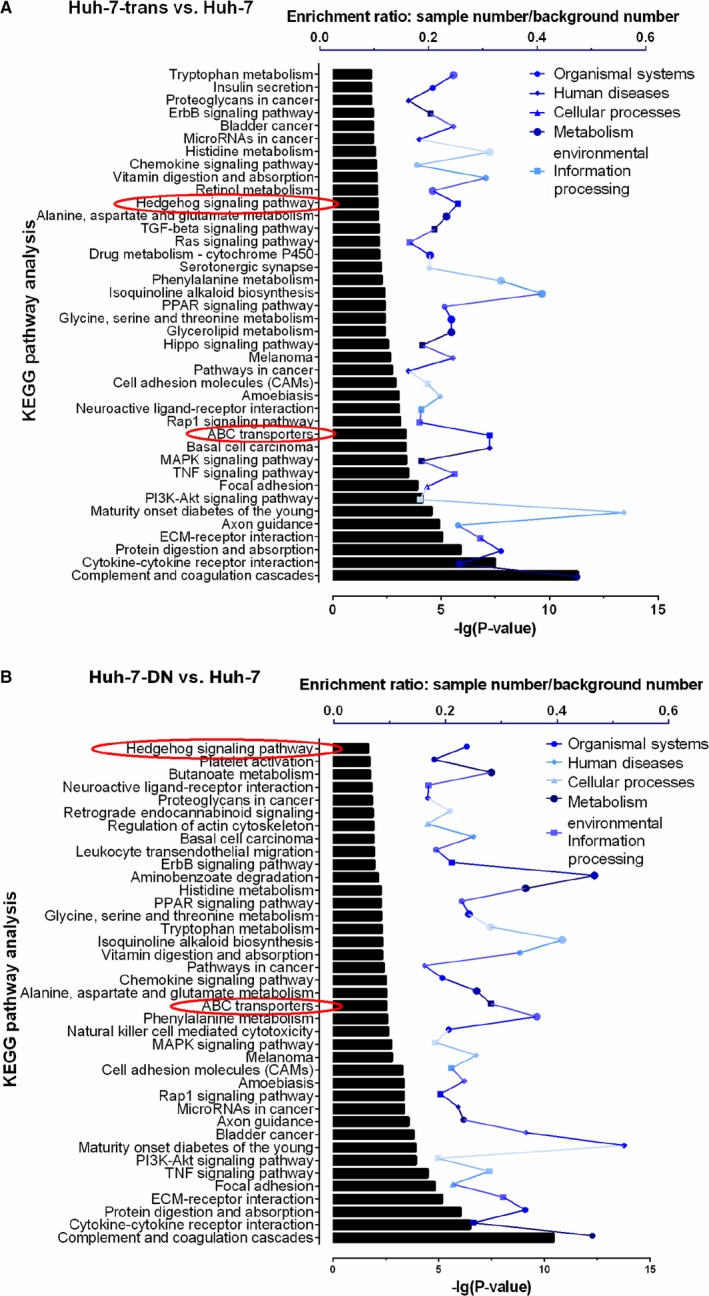
ABC transporters and hedgehog signalling pathway were identified in enrichment through KEGG pathway analysis from the data of Huh‐7‐trans and Huh‐7‐DN vs Huh‐7 cells after an RNA‐sequencing assay. Enrichment ratio was showed by sample number/background number. Sample number: quantity of differential genes in relative KEGG pathway, background number: total gene quantity in relative KEGG pathway of Homo sapiens. Correlated *P* value ≤ .05 was considered for KEGG enrichment. Top 40 of enrichment was listed. KEGG, Kyoto Encyclopaedia of Genes and Genomes

Subsequent quantitative RT‐PCR confirmed that compared with parental Huh‐7 cells, Huh‐7‐trans and Huh‐7‐DN subpopulations lost mature hepatocyte‐specific biomarkers (AFP, CEBPA, CYP2C9, UGT1A1, GSTA1/2) and significantly increased expression of cancer progression‐associated genes (FGF1/5, PDGFB, PDGFRB, TGFB1, VEGFC, WNT5B), indicating that these two subpopulations were poorly differentiated. Despite largely overlapped, the differential rate of gene expression between Huh‐7‐trans and Huh‐7‐DN cells was of 1.2%, and the difference mainly exists in metastatic and drug‐resistant features. Gene expression of EMT markers (MMP1, S100A4 and TWIST1) was markedly higher in Huh‐7‐trans cells than Huh‐7‐DN cells, and an ABC transporter member, ABCG4 was significantly increased in Huh‐7‐DN cells in consistence with its chemoresistant feature (Figure 2C‐N and Figure S1). KEGG analysis was used to identify the related differential pathway profiles in Huh‐7‐trans and Huh‐7‐DN subpopulations vs Huh‐7 cells. The top 40 enriched differentially KEGG terms in Huh‐7‐trans and Huh‐7‐DN subpopulations were listed in Figure 3A,B. Hedgehog signalling pathway and ABC transporters were both significantly enriched in Huh‐7‐trans and Huh‐7‐DN subpopulations. The enrichment ratio, identified by sample number/background number, is 0.3125 of ABC transporter and 0.2540 of Hedgehog signalling pathway in Huh‐7‐trans subpopulations vs Huh‐7 cells while 0.2813 and 0.2381 of that in Huh‐7‐DN subpopulations vs Huh‐7 cells, respectively.

### 3.3 Expression of Hh transcription factor GLI1 and TAP1 in HCC tissues

**Figure 4:**
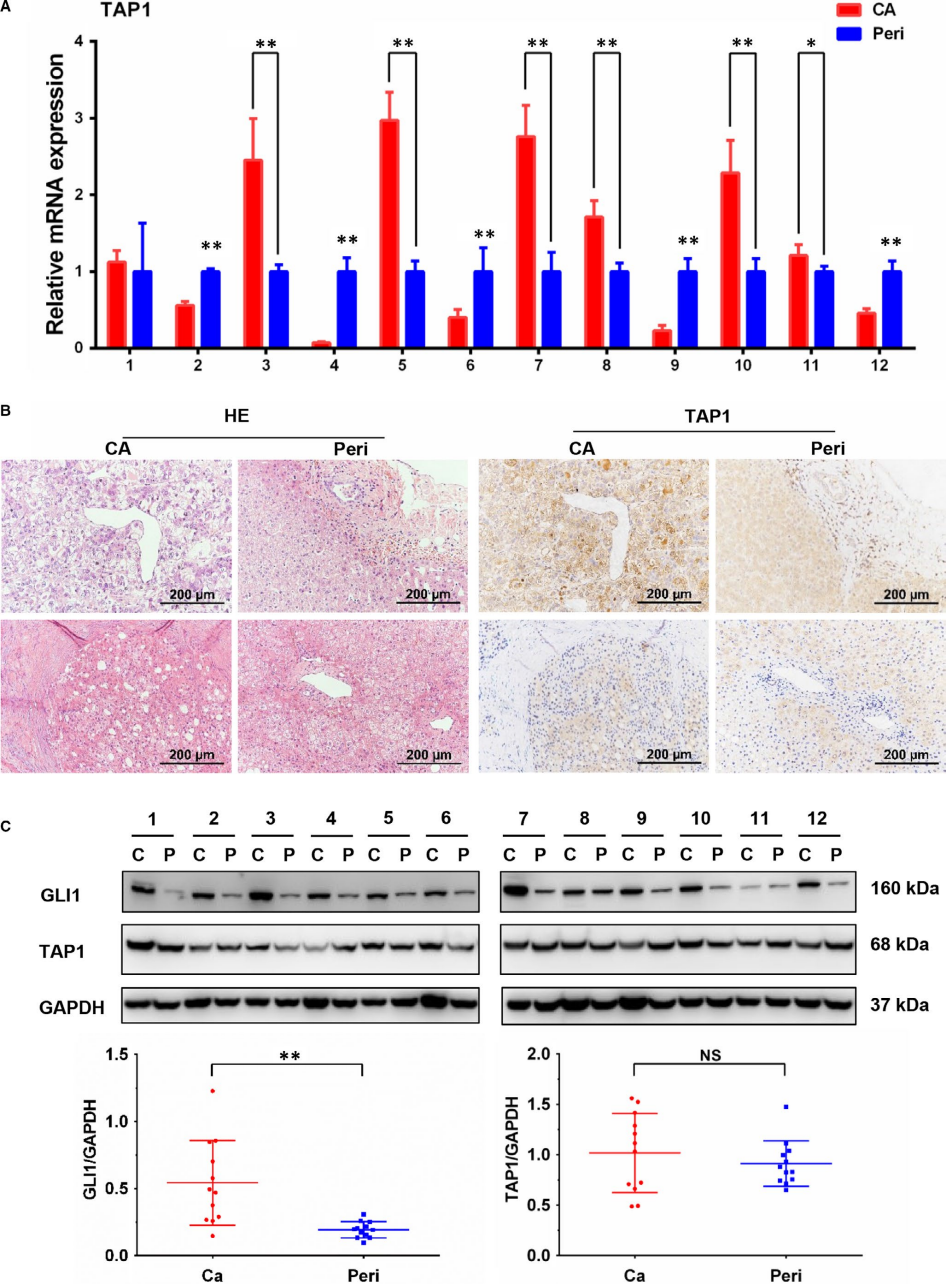
Hedgehog signalling activation and TAP1 expression in HCC specimens. A, Relative expression of TAP1 in HCC specimens and corresponding pericancerous tissue was determined by quantitative RT‐PCR (n = 12). *P < .05; **P < .01 compared to pericancerous tissue. B, Representative images (upper: #5, bottom: #10) of H‐E staining (left) and TAP1 expression (right) were acquired by inverted microscope (Scale bar = 200 μm). C, Western blot analysis of GLI1 and TAP1 in HCC specimens and corresponding pericancerous tissue and quantification of GLI1 and TAP1 protein expression after normalization for loading controls (GAPDH). GLI1 polyclonal antibody (Novus Biologicals) was used for blotting. n = 12, *P < .05, **P < .01 compared with corresponding pericancerous tissue

To assess the clinical relevance between the hedgehog signalling activation and TAP1, expression of GLI1 and TAP1 was detected in 12 pairs of HCC specimens and pericancerous tissues, including 8 HBV‐associated HCC, 1 fatty liver‐associated HCC and 3 cases with unknown base disease. There are 8 patients with increased AFP levels and 4 patients with normal values (AFP < 10 μg/L prior to surgical resection). Among these patients, 8 out of 12 (75%) HCC specimens had significantly increased GLI1 protein levels. It is worth to mention that GLI1 protein levels were all significantly higher in HCC specimens with a normal serum AFP value (#3, 7, 10, 12) than their pericancerous tissue. An increase in TAP1 gene expression was observed in 6 out of 12 (50%) HCC cases. Three of them had AFP with normal value (#3, 7, 10) (Table S5). Immunohistochemical staining showed that six specimens with increased TAP1 mRNA levels exhibited moderate to strong TAP1 expression in the cytoplasm of malignant cells (Figure 4A,B). Consistent with qRT‐PCR and immunohistochemical staining, TAP1 protein levels significantly increased in 3 out 12 of HCC specimens accompanied with an increase in GLI1 protein (Figure 4C). In summary, hedgehog signalling activation is common in HCC tissues; whereas TAP1 is heterogeneously expressed. Both GLI1 and TAP1 tend to increase in HCC specimens with normal serum AFP value.

### 3.4 GLI1 mediates TAP1 expression in poorly differentiated hepatoma cells

**Figure 5:**
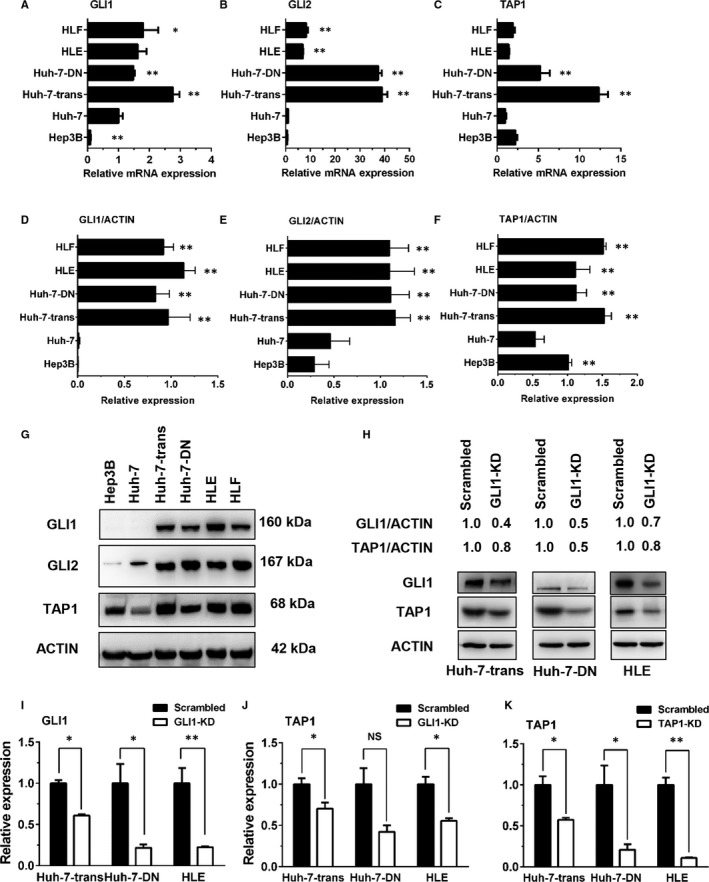
Hedgehog signalling activation and TAP1 expression in hepatoma cells. Relative mRNA expression of GLI1 (A), GLI2 (B) and TAP1 (C) in Hep3B, Huh‐7, Huh‐7‐trans, Huh‐7‐DN, HLE and HLF cell lines was determined by quantitative RT‐PCR. Western blot analysis of GLI1, GLI2 and TAP1 in hepatoma cell lines and relative expression of GLI1 (D), GLI2 (E) and TAP1 (F) after normalization for ACTIN as a loading control. GLI1 monoclonal antibody (Cell Signaling Technology) was used for blotting. G, n = 3 *P < .05; **P < .01. H, GLI1 and TAP1 protein levels in GLI1‐KD Huh‐7‐trans, Huh‐7‐DN and HLE cells by Western blot analysis. Desitometric ratios of GLI1 and TAP1 over β‐actin were compared with those transduced with lentivirus harbouring scrambled shRNA controls (set to 1.0). Relative mRNA expression of GLI1 (I) and TAP1 (J) in GLI1‐KD Huh‐7‐trans, Huh‐7‐DN and HLE cells compared with those transduced with lentivirus harbouring scrambled shRNA controls was determined by quantitative RT‐PCR. Relative mRNA expression of TAP1 (K) in TAP1‐KD Huh‐7‐trans, Huh‐7‐DN and HLE cells compared with those transduced with lentivirus harbouring scrambled shRNA controls were determined by quantitative RT‐PCR. GLI1‐KD cells: GLI1 was knocked‐down through lentiviral transduction; TAP1‐KD cells: TAP1 was knocked‐down through lentiviral transduction; Scrambled: scrambled shRNA control was transduced though lentiviral transduction. n = 3, *P < .05 compared to scrambled shRNA controls

Given that enhanced hedgehog signalling activation and elevated TAP1 mRNA expression were found in poorly differentiated Huh‐7‐trans, Huh‐7‐DN cells and HCC specimens with lower AFP levels, their expression in a variety of hepatoma cell lines with different differentiation degree was determined to investigate whether this phenomenon is universal in poorly differentiated hepatoma cells. The results showed that mRNA levels of Hh transcription factors GLI1 and GLI2 were obviously increased in poorly differentiated Huh‐7‐trans, Huh‐7‐DN, HLE and HLF cells compared with well‐differentiated Hep3B and Huh‐7 cells. Consistent with their mRNA levels, GLI1 and GLI2 protein levels were significantly increased in these four poorly differentiated hepatoma cell types. Along with the Hh signalling activation, TAP1 protein levels in these hepatoma cell types were significantly higher than Huh‐7 cells (Figure 5A‐G). These findings suggested that the Hh signalling activation may contribute to TAP1 expression in poorly differentiated hepatoma cells.

### 3.5 Suppressing GLI1 by an RNAi approach led to decreased TAP1 expression

Huh‐7‐trans, Huh‐7‐DN and HLE cells were transduced by lentiviral vectors harbouring shRNAs against GLI1 or TAP1. GLI1 and TAP1 protein levels were significantly down‐regulated in these three cell types after lentiviral transduction with specific shRNAs compared with those transduced with lentiviral vector harbouring scrambled shRNA control. Moreover, GLI1 knock‐down with specific shRNA caused a significant decrease in TAP1 protein levels of all three cell types, indicating that TAP1 expression may be under the control of Hh transcription factor GLI1 (Figure 5H‐K).

### 3.6 Inhibition of Hh signalling restored chemosensitivity in poorly differentiated hepatoma cells

**Figure 6:**
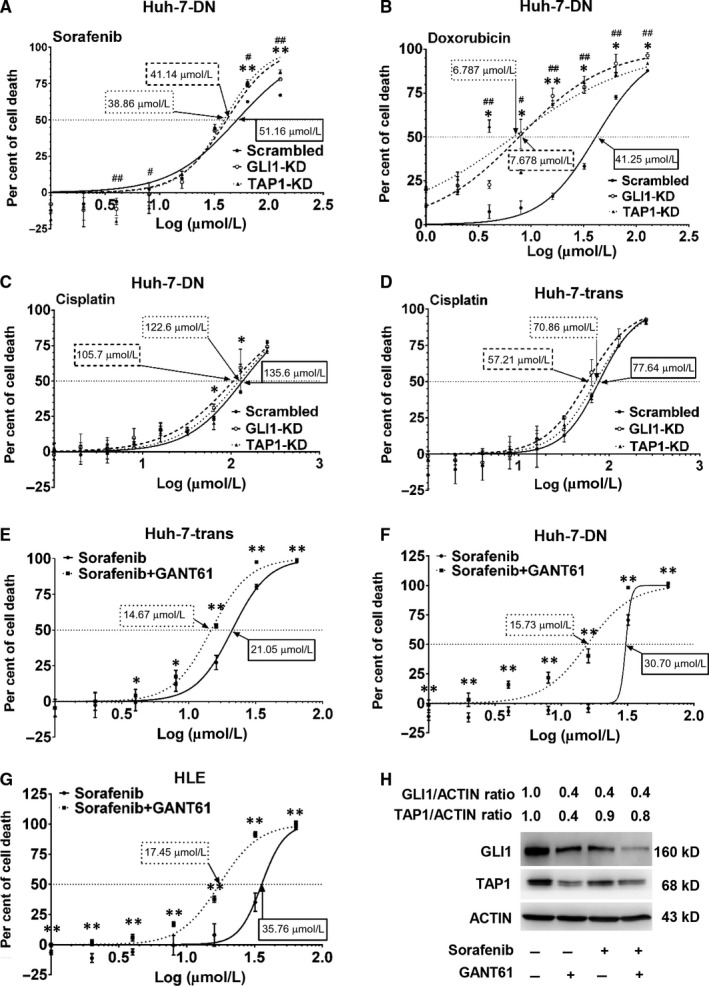
Chemosensitivity of Huh‐7‐trans and DN cells exposed to sorafenib, doxorubicin or cisplatin after blocking Hh signalling. Chemosensitivity of TAP1‐KD and GLI1‐KD of Huh‐7‐DN cells exposed to Sorafenib (A), doxorubicin (B) or cisplatin (C) and viability of TAP1‐KD and GLI1‐KD of Huh‐7‐trans cells exposed to cisplatin (D) was determined by MTT assay. n = 3, *P < .05; **P < .01; GLI1‐KD cells compared with scrambled shRNA controls. #*P* < .05; ##*P* < .01; TAP1‐KD cells compared with scrambled shRNA controls. Chemosensitivity of Huh‐7‐trans (E), Huh‐7‐DN (F) and HLE cells (G) exposed to Sorafenib with/without 5 μmol/L GANT61. n = 3, *P < .05; **P < .01 compared with Sorafenib monotherapy. H, GLI1 and TAP1 expression in HLE cells treated with/without Sorafenib (16 μmol/L) or GANT61 (5 μmol/L) by Western blot analysis. GLI1 monoclonal antibody (Cell Signaling Technology) was used for blotting. Desitometric ratios of GLI1 and TAP1 over β‐actin were compared with HLE cells without any treatment (set to 1.0). n = 3

To evaluate whether hedgehog signalling pathway mediates drug sensitivity through TAP1 in poorly differentiated hepatoma cells, Huh‐7‐trans and Huh‐7‐DN with GLI1‐KD or TAP1‐KD were treated with Sorafenib, doxorubicin or cisplatin at a concentration gradient for 24 hours. Compared with those transduced with lentiviral vector harbouring scrambled shRNA controls, IC50 of Sorafenib in Huh‐7‐DN cells with GLI1‐KD or TAP1‐KD was reduced from 51.16 to 41.14 μmol/L and 38.86 μmol/L, respectively. Similarly, IC50 of doxorubicin in Huh‐7‐DN cells with GLI1‐KD or TAP1‐KD was significantly decreased from 41.25 to 7.68 μmol/L and 6.79 μmol/L, separately. Both Huh‐7‐trans and Huh‐7‐DN cells with GLI1‐KD and TAP1‐KD treated with cisplatin had worsening cell viability in comparison with those transduced with scrambled shRNA controls (Figure 6A‐D). Moreover, three poorly differentiated hepatoma cell types were further treated with a GLI antagonist, GANT61 at 5 μmol/L in combination with Sorafenib, doxorubicin or cisplatin for 24 hours. A combined treatment of GANT61 with Sorafenib resulted in a remarkable decrease in IC50 of all three hepatoma cell types, from 21.05 to 14.67 μmol/L in Huh‐7‐trans cells, 30.70 to 15.73 μmol/L in Huh‐7‐DN cells, and 35.76 to 17.45 μmol/L in HLE cells (Figure 6E‐G). A similar effect was observed when GANT61 was combined with doxorubicin or cisplatin in these three cell types (Figure S2). Given that GANT61 inhibited GLI1/2 nuclear accumulation and transacting activity, both GLI1 and TAP1 protein levels were decreased in HLE cells in exposure to GANT61 in consistent with decreased TAP1 expression by shGLI1 knock‐down (Figure 6H). These data suggest that GLI1 could be an upper stream regulator of TAP1, and suppression of hedgehog transcription factors GLI1 by either GANT61 or RNAi ameliorated chemo‐resistance in poorly differentiated hepatoma cells.

### 3.7 GLI1 transactivates TAP1 expression in poorly differentiated hepatoma cells

**Figure 7:**
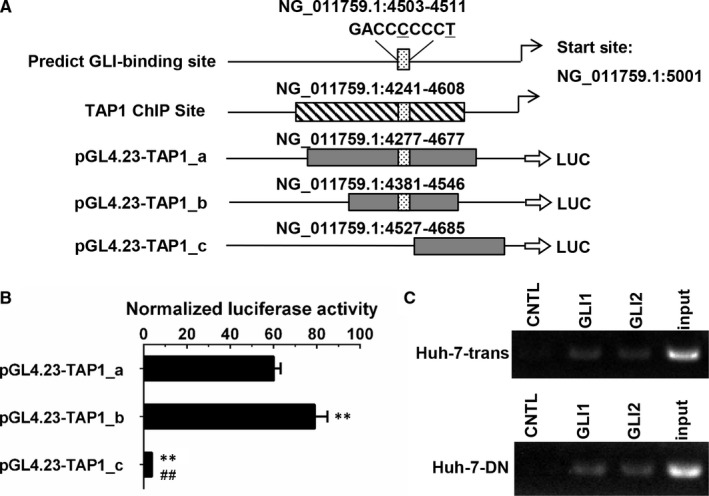
Dual‐luciferase reporter assay and chromatin immunoprecipitation assay of GLI1/2‐binding site in the TAP1 promoter region. A, A diagram depicts ChIP‐binding site in the TAP1 promoter regions. Two sequences containing potential binding site and one sequence lacking the putative site were inserted into luciferase reporter plasmid pGL4.23. The ChIP site and predicted non‐consensus GLI‐binding site are indicated by box with slash and box with dot, respectively. Two site variants in position 5 and position 9 are indicated with underline. B, Relative luciferase ratio of cotransfected pGL4.23‐TAP1_a, pGL4.23‐TAP1_b or pGL4.23‐TAP1_c construct with pRL‐TK vector in HLE cells. C, Chromatin immunoprecipitation assay showed GLI1 and GLI2 bound to the promoter region of TAP1 in both Huh‐7‐trans cells (upper) and Huh‐7‐DN cells (bottom). CNTL, negative control. **P < .01 vs pGL4.23‐TAP1_a; ##*P* < .01 vs pGL4.23‐TAP1_b

Having shown that GLI1 may be an upper stream regulator of TAP1, we further determine whether TAP1 is a direct transcriptional target of GLI1. Hedgehog transcription factors GLI1/2 bind to a conserved sequence 5′‐GACCACCCA‐3′ to initiate the transcription of target genes, and a potential GLI‐binding sequence 5′‐GACCCCCCT‐3′ (NG_011759.1:4503‐4511) located at 500 bp proximal to the transcription starting site in the TAP1 promoter was predicted by MatInspector program. To validate its functionality, we designed two constructs of pGL4.23‐TAP1_a & b containing the predicted GLI‐binding sequence, and pGL4.23‐TAP1_c construct lacking the putative binding site (Figure 7A). Three luciferase‐reporter constructs containing predicted sequences or lacking the potential sequence were transfected into HLE cells which displayed highest transfection efficiency in three hepatoma cell types, and pRL‐TK renilla luciferase vector was cotransfected for the normalization of transfection efficiency. Normalized luciferase ratio of pGL4.23‐TAP1_a/b constructs was increased more than 18‐fold compared with pGL4.23‐TAP1_c construct 48 hours after transfection (Figure 7B). These results demonstrate that pGL4.23‐TAP1_a/b does contain the binding sequence in the TAP1 promoter. Furthermore, ChIP assay validated that after immune precipitation with antibodies against GLI1 or GLI2, the potential GLI‐binding sequence within the TAP1 promoter could be amplified and visualized in gel of PCR products from the precipitated DNA, and it appears to be that the GLI1/2 transcription factors elicit TAP1 transcription when they bind to the putative sequence in poorly differentiated hepatoma cells (Figure 7C).

## 4 DISCUSSION

Drug resistance is a critical issue in improving outcome of chemotherapy in HCC. Two medications in the first line: Sorafenib, a multikinase inhibitor and Lenvatinib, a multitarget tyrosine kinase VEGFR and FGFR inhibitor, are currently used for the treatment of advanced HCC.^8^, ^9^, ^10^ Possible mechanisms inducing resistance to Sorafenib are thought to be autophagy and other pathways^11^, ^12^; however, precise mechanisms remain elusive. First‐line and second‐line chemotherapeutic agents, such as Lenvatinib, Pembrolizumab, Ramucirumab, Regorafenib and Cabozantinib, have been approved in an accelerated rate by the FDA in the latest 3 years.^13^, ^14^, ^15^, ^16^, ^17^, ^18^ Therefore, there are more choices for systemic drug‐therapy for advanced HCC compared with the situation of Sorafenib mono‐availability before 2017. Nevertheless, the improvement in overall survival has not been satisfactorily achieved, and objective responsive rate is far to be increased with molecular‐targeting therapeutics or check‐point inhibitors. To approach overwhelming insensitivity of HCC to chemotherapeutics, our initial objective is to investigate the intrinsic mechanism of chemo‐resistance in advanced HCC and to develop novel approaches to interrupt specific pathways that are critical in the modulation of drug resistance.

In our previous studies, Huh‐7‐trans and Huh‐7‐DN, two subpopulations derived from Huh‐7 cells with double negative surface markers CD133 and EpCAM exhibited multidrug resistance against Sorafenib and Itraconazole.^4^, ^5^ We further characterized their cytogenetic and phenotypic features by karyotypic analysis and transcriptome profiling. As expected, abnormal karyotypic features were found in Huh‐7 cells; Huh‐7‐trans and Huh‐7‐DN cells had a higher frequency of cytogenetic abnormalities than Huh‐7 cells. These distinct alterations documented that both Huh‐7‐trans and Huh‐7‐DN are two particular subpopulations with characteristic karyotypic features. These cytogenetic abnormalities may well be responsible for their aggressive behaviours of metastasis, progression and drug resistance. However, exact molecular cascades from aberrant karyotypic control to phenotypic molecular interplays remain unexplored.

To uncover the molecular interplays of drug resistance in Huh‐7‐derived subpopulations, RNA‐sequencing analysis was used to determine the transcriptome as a snapshot of differential gene expression profile. It is apparent from the heat map of the verified data set that major differentially expressed genes that are categorized into Hh (GLI1, GLI2), Wnt (WNT5B) or Hippo signalling (FGF1, TGFB1) are involved in the modulation of cancer progression and were up‐regulated, indicating that Huh‐7‐trans and Huh‐7‐DN cells might be more aggressive than parental Huh‐7 cells. Moreover, Huh‐7‐trans and Huh‐7‐DN cells were found to over‐express a variety of genes associated with EMT (MMP1, S100A4, SERPINE1, TWIST1), ABC transporter, stemness (SOHLH2); and down‐regulated expression of hepatocyte‐specific genes (AFP, CEBPA, CYP2C9, UGT1A1, GSTA1 and GSTA2), indicating that two subpopulations were highly de‐differentiated with overt metastatic and drug‐resistant properties. Furthermore, KEGG analysis discovered that both ABC transporter and Hh signalling pathway were significantly enriched in Huh‐7‐trans and Huh‐7‐DN vs parental Huh‐7 cells, implicating that the ABC transporter and Hh signalling could be key pathways in the mediation of drug resistance in both Huh‐7‐trans and Huh‐7‐DN subpopulations.

From surgically resected HCC specimens, it is clear that Hh signalling was activated, since GLI1 was up‐regulated in approximately 75% in HCC specimens, indicating that the Hh signalling might play a pivotal role in oncogenesis or progression. In our previous study, we found that ABCC1 and Hh transcription factor GLI2 were up‐regulated in Huh‐7‐DN cells, which displayed profound resistance to chemotherapy drugs, indicating GLI2 could regulate ABCC1 to increase drug resistance.^5^ However, Huh‐7‐trans cells were also found to be drug‐resistant in addition to their highly metastatic property. In order to narrow down which genes are fundamental for drug resistance in advanced HCC, we screened genes belonging to ABC transporters, which were reported to function as efflux molecules of anticancer drugs^19^ or bile acid transporters, and may be involved in cholestasis^20^ and other molecular processes. In this study, TAP1 was up‐regulated at mRNA and protein levels in both Huh‐7‐trans and Huh‐7‐DN cells and its expression was confirmed to be higher in two subpopulations than other hepatoma cell lines. Quantitative RT‐PCR and Western blot analyses further validated that TAP1 was up‐regulated in Huh‐7‐trans and Huh‐7‐DN cells, so were GLI1/2, although there is significant heterogeneity in terms of TAP1 expression in HCC specimens. These findings were in the line with an early report that demonstrated TAP1 as a critical factor in the mediation of drug resistance by SHH in pancreatic ductal adenocarcinoma.^21^ The higher frequency of increased TAP1 levels in HCC specimens with a normal serum AFP level may indicate that TAP1 expression is associated with poorly differentiated pathologic classification (all in II‐III classes), although a large number of HCC specimens are needed to allow such a correlation analysis along with other markers, such as glypican‐3 (GPC‐3), (Table S5) currently used in clinics for HCC detection.

To confirm the relationship between hedgehog signalling and TAP1 expression, molecular mechanism of the Hh signalling in regulating TAP1 expression was determined by lentiviral transduction‐mediated RNAi, dual‐luciferase reporter and chromatin immunoprecipitation assays. The results indicate that GLI1/2 may bind to the consensus sequence of the TAP1 promoter region and regulate expression of the downstream target genes, including TAP1.^22^ To confirm the key role of TAP1 during drug resistance, inhibition curves of Huh‐7‐trans and Huh‐7‐DN cells with lentivirus‐mediated GLI1 or TAP1 knocked‐down in exposure to different therapeutic drugs were established. As shown in the inhibition curves, knocking‐down of either GLI1 or TAP1 or use of a GLI1 inhibitor, GANT61, sensitized Huh‐7‐DN cells to doxorubicin toxicity, indicating that TAP1 may be a critical molecule in the mediation of drug resistance in both Huh‐7‐trans and Huh‐7‐DN cell types. In this context, both hedgehog signalling pathway and TAP1 could be valuable molecular targets for improving the outcome of chemosensitivity of a poorly differentiated type of HCC, which features with often both CD133‐ and EpCAM‐negative, exhibits marked EMT properties and possesses a devastating prognosis.^3^, ^4^

In conclusion, both Huh‐7‐trans and Huh‐7‐DN are two distinct subpopulations sharing many cytogenetic and molecular similarities with parental Huh‐7 cells, and worsening abnormality in karyotypic features and differential gene expression profiles are highly responsible for their phenotypic malignancy, aggressive metastatic and drug‐resistant behaviours. TAP1 may account for drug resistance in both Huh‐7‐trans and Huh‐7‐DN cell types under the transcriptional control of GLI1/2. Suppressing GLI1 or TAP1 gene expression ameliorates drug resistance in hepatoma cells. Taken together, these findings confer novel insights into improving the treatment outcome of refractory HCC by targeting the hedgehog signalling pathway and TAP1 molecule.

## CONFLICT OF INTEREST

The authors declare no conflict of interest associated with this study.

## AUTHOR CONTRIBUTIONS

X‐T Z, J D and H‐Y L performed the research; X‐T Z, J D and J W designed the research study; J‐L Z, S‐Y G and H‐L J contributed to clinical human samples; X‐T Z and J D analysed the data and wrote the paper; J D, H‐L J and J W revised the paper critically. All authors read and approved the final version of the manuscript.

## Data Availability

The data used to support the findings of this study are available from the corresponding authors upon request.
