# Supplementary material for: Hedgehog signalling mediates drug resistance through targeting TAP1 in hepatocellular carcinoma
Source: J Cell Mol Med. 2020 Feb 28;24(7):4298–311. doi: 10.1111/jcmm.15090 (PMC7171417; doi:10.1111/jcmm.15090)

Hedgehog Signaling Mediates Drug Resistance through Targeting TAP1 in Hepatocellular Carcinoma

Xiao-Tian Zhou[1], Jia Ding[2*], Hui-Yan Li[1], Jie-Liang Zuo[3], Sheng-Yang Ge[3], Hu-Liang Jia[3*], Jian Wu[1,4,5*]

[1]Department of Medical Microbiology and Parasitology, MOE/NHC/CAMS Key Laboratory of Medical Molecular Virology, School of Basic Medical Sciences, Shanghai Medical College, Fudan University, Shanghai 200032, China

[2]Department of Gastroenterology, Shanghai Jing'an District Central Hospital, Fudan University, Shanghai 200040, China

[3]Department of General Surgery, Huashan Hospital of Fudan University, Shanghai 200040, China

[4]Department of Gastroenterology & Hepatology, Zhongshan Hospital of Fudan University, Shanghai 200032, China

[5]Shanghai Institute of Liver Diseases, Shanghai Medical College, Fudan University, Shanghai 200032, China

***Corresponding Authors:**

Jian Wu, MD, PhD, FAASLD

Email: jian.wu@fudan.edu.cn

Jia Ding, MD, PhD

Email: jiading12@fudan.edu.cn

Huliang Jia, MD, PhD

Email: jbl-1@163.com

SUPPLEMENTAL INFORMATION

RESULTS

Inhibition of Hh transcription factors GLI1/2 improved drug sensitivity in poorly-differentiated hepatoma cells

Given the fact that Hh signaling transcription factor GLI2 regulates drug sensitivity through ABCC1 transporter in our previous study [1], three hepatoma cells were treated with a GLI antagonist, GANT61, at 1.25, 2.5, 5, 10, 20, 40 μM for 24 hrs to inhibit transcription activity of GLI1/2. As shown in **Suppl. Fig. 2A-C**, GANT61 at 5 μM did not affect cell viability of Huh-7-trans, Huh-7-DN and HLE cells. A combined treatment of GANT61 at 5 μM with doxorubicin resulted in 25~30 % decrease in viability of all three hepatoma cells, and IC50 was remarkably shifted from 80.98 to 6.41 μM in Huh-7-trans cells, 50.84 to 6.05 μM in Huh-7-DN cells, and 30.74 to 8.19 μM in HLE cells. Compared to cisplatin monotherapy, combination of GANT61 with Sorafenib led to a significant decrease in viability, and corresponding IC50 was diminished from 81.36 to 41.39 μM in Huh-7-trans cells, 113.3 to 25.92 μM in Huh-7-DN cells, and 82.47 to 43.37 μM in HLE cells (**Suppl. Fig. 2D-I**).

REFERENCES

1. **Ding J, Zhou XT, Zou HY, *et al.*** Hedgehog signaling pathway affects the sensitivity of hepatoma cells to drug therapy through the ABCC1 transporter. *Lab Invest* 2017;97:819-832.
2. **Scheuer PJ.** Classification of chronic viral hepatitis: a need for reassessment. *J Hepatol* 1991;13:372-374.
3. **Edmondson HA, Steiner PE.** Primary carcinoma of the liver: a study of 100 cases among 48,900 necropsies. *Cancer* 1954;7:462-503.

SUPPLEMENTARY TABLES

Supplementary Table 1 Primer sequences for Quantitative RT-PCR

GENE	Forward Sequence (5'-3')	Reverse Sequence (5'-3')	Length (bp)
ABCG4	ATCTTGGCAGGATAC	TAGCAGGACATCTTG	95
AFP	CTTTGGGCTGCTCGCTATGA	GCATGTTGATTTAACAAGCTGCT	131
BCL2	TGTGTGGAGAGCGTCAAC	GTTCAGGTACTCAGTCATCC	75
CEBPA	CATCTGCGAGCACGAGAC	AGGAACTCGTCGTTGAAG	72
CYP2C9	ACAACCAACCATCTGAAT	AGGAGAAGGAGAGCATAT	112
FGF1	TTCACAGCCCTGACCGAGAA	CGTTGCTACAGTAGAGGAGTTTG	76
FGF5	CACTGATAGGAACCCTAGAGGC	CAGATGGAAACCGATGCCC	196
GAPDH	GAAGATGGTGATGGGATTTC	GAAGGTGAAGGTCGGAGTC	226
GLI1	ACAGAAGGACTGTCTGGCCCGC	TCGGTGCAGCTGTTGGTCTC	98
GLI2	GTAAGCAGGAGGCTGAGGTG	GGATGTGCTCGTTGTTGATG	114
GSTA1	CTGCCCGTATGTCCACCTG	AGCTCCTCGACGTAGTAGAGA	185
GSTA2	CTCCACTACTCCAATATAC	CCATCAATCTCAACCATT	167
MMP1	AAAATTACACGCCAGATTTGCC	GGTGTGACATTACTCCAGAGTTG	82
PDGFB	GCGGAAGAAGCCAAT	CTGCCACTGTCTCAC	77
PDGFRB	AGACACGGGAGAATACTTTTGC	AGTTCCTCGGCATCATTAGGG	126
S100A4	CCTGGATGTGATGGTGTC	CTCCTTTAGTTCTGACTTGTTG	85
SERPINE1	ACCGCAACGTGGTTTTCTCA	TTGAATCCCATAGCTGCTTGAAT	109
SOHLH2	GCTTCCTCAATTATCTGCCAGG	CCCACAGTGACATCTCCAACT	86
TAP1	CGCCTCACTGACTGGATTCTA	TCTGTTGGAAAAACTCCGTCTC	208
TGFB1	CTAATGGTGGAAACCCACAACG	TATCGCCAGGAATTGTTGCTG	209
TWIST1	GTCCGCAGTCTTACGAGGAG	GCTTGAGGGTCTGAATCTTGCT	156
UGT1A1	CATGCTGGGAAGATACTGTTGAT	GCCCGAGACTAACAAAAGACTCT	214
VEGFC	GAGGAGCAGTTACGGTCTGTG	TCCTTTCCTTAGCTGACACTTGT	96
WNT5B	GCTTCTGACAGACGCCAACT	CACCGATGATAAACATCTCGGG	77

Supplementary Table 2 Primary antibodies for Western blot assay, IHC and ChIP

Antibody	Company	Application
ACTIN	Proteintech, Wuhan, Hubei, China	Western blot
GAPDH	Proteintech, Wuhan, Hubei, China	Western blot
GLI1	Cell Signaling Technology, Danvers, MA, USA	ChIP & Western blot (for cell experiments)
GLI1	Novus Biologicals, Centennial, CO, USA	Western blot (for HCC specimens)
GLI2	Abcam, Cambridge, UK	ChIP
GLI2	Novus Biologicals, Centennial, CO, USA	Western blot
TAP1	Abcam, Cambridge, UK	Immunohistochemical staining
TAP1	Cell Signaling Technology, Danvers, MA, USA	Western blot

Supplementary Table 3 shRNA sequences

GENE	Sense sequence (5’-3’)	Antisense sequence (5’-3’)	Region
TAP1	CCGTGTGTACTTATCCTGGAT	ATCCAGGATAAGTACACACGG	CDS
GLI1	CCTGATTATCTTCCTTCAGAA	TTCTGAAGGAAGATAATCAGG	CDS
Scrambled	CCTAAGGTTAAGTCGCCCTCG	CGAGGGCGACTTAACCTTAGG	

CDS, Coding sequence.

Supplementary Table 4 Primer sequences of putative binding sequence of GLI1/2 within the TAP1 promoter incorporation into the reporter system

TAP1 promoter constructs	Primer sequences (5'-3')	Product position and size
pGL4.23-TAP1_a	F: TTCCTTCTCCCAAGTGCTGTTC	NG_011759.1:4277-4677
	R: GGGTCTGGGCTTGAGGGTT	(401 bp)
pGL4.23-TAP1_b	F: GGGACCAGAGTGAAAGCGAAAG	NG_011759.1:4381-4546
	R: CAACCCGCAATGAGCATAGAGTA	(166 bp)
pGL4.23-TAP1_c	F: TCTATGCTCATTGCGGGTTGC	NG_011759.1:4527-4685
	R: GGGTAATGGGTCTGGGCTTG	(159 bp)

Supplementary Table 5 Demographic information of HCC patients

Experimental #	1	2	3	4	5	6	7	8	9	10	11	12
Gender	M	M	F	M	M	F	F	M	M	M	M	M
Age	54	60	61	63	69	75	75	51	47	61	57	71
Alcohol Intake	Y >150 g/d for 25 yr	N	N	N	N	N	N	N	N	N	N	N
Base Liver Disease [a]	CHB -G2S4	CH -G1S4	CHB -G1S4	CHB -G1S0	CHB -G1S4	CH -G0S0	G2S4	CHB -G3S4	CHB -G0S3	Hepatic steatosis (33-66%)	CHB -G2S4	CHB -G2S1
Clinical Diagnosis	Liver cancer	Liver cancer	Liver cancer	Liver cancer	Relapse post -HCC surgery	Post HCC surgery and TACE	Relapse post -HCC surgery	Liver cancer, cirrhosis post CHB	Liver cancer	Liver cancer	Relapse post -HCC surgery	Relapse post -HCC surgery
TNM Classification	T1N0 M0	T2N0 M0	T1N0 M0	T2N0 M0	T2N0 M0	T2N0M0	T2N0 M0	T4N0M0	T2bN 0M0	T1N0M0	T4NXM0	T1N0 M0
Pathologic Diagnosis	HCC	HCC	HCC	HCC	HCC	HCC	HCC	HCC	HCC	HCC	HCC	HCC
Histopathologic Grade [b]	III	II	II	III-IV	III	II	II	II	II	II	III	III
MVI	M2	M2	M0	M1	M2	M1	M1	M2	M1	M0	M2	M3
Within Hepatic Metastasis	N	Y	N	N	Y	Y	N	N	N	N	Y	N
Portal Vein Invasion (Y/N)	Y	Y	N	Y	Y	Y	Y	Y	Y	N	Y	Y
Adjacent Tissue Invasion	N	N	N	N	N	Y	N	N	N	N	Y	N

**Experimental #**		**1**	**2**	**3**	**4**	**5**	**6**	**7**	**8**	**9**	**10**	**11**	**12**
Invasion Organ							Adrenal gland					Diaphragm	
Distal Organ Metastasis		N	N	N	N	N	N	N	N	N	N	N	N
Relapsed (y/n)		N	N	N	N	Y	Y	Y	N	N	N	Y	Y
Secondary Resection (Y/N)		N	N	N	N	Y	N	Y	N	N	N	Y	Y
AFP (ng/ml)	<10	302.58	223.5	1.82	185	3184.9	466.2	2.57	4502.7	1430.5	2.1	620	1.94
HBsAg		+	-	+	+	+	-	+	+	-	-	+	+
HBsAb		-	-	-	+	+	-	-	-	-	+	-	-
HBeAg		-	-	-	-	-	-	-	-	-	-	+	-
HBeAb		+	+	-	-	+	+	+	+	+	-	-	+
HBcAb		+	+	+	+	+	+	+	+	+	+	+	+
HCV-Ab		-	-	-	-	-	-	-	-	-	-	-	-
HBV-DNA		/	/	/	U[c]	U	U	U	U	U	U	U	U
Staining of Resected Specimens	CK18	+	+	+	+	+	+	+	+	+	+	+	+
	CK19	+	-	-	focal+	+	/	-	few+	-	Cholangio +	-	-
	HEP1	-	+	few+	-	-	-	+	-	+	+	+	+
	GPC-3	+	+	+	+	+	/	-	+	-	-	+	+

Footnotes:

a. Base liver disease: Scheuer scoring system [2], CHB: Chronic Hepatitis B. G: for necroinflammatory activity in chronic hepatitis; S: scoring system for fibrosis and cirrhosis.

b. Histopathologic Edmondson-Steiner grade [3].

c. U: under detectable limit.

TACE= transarterial chemoembolization

MVI = Microvascular invasion

GPC-3 = Glypican-3

Hep1= Hepatocyte paraffin 1

CK18 = Cytokeratin 18

CK19 = Cytokeratin 19

TNM = Tumor, Nodes, Metastasis

SUPPLEMENTARY FIGURE LEGENDS

Supplementary Fig. 1 Representative of differential gene expression levels of hepatocyte-specific genes (**A, B**), drug-resistance (**C**) and cancer progressive genes (**D-H**) verified by quantitative RT-PCR using Huh-7 cells as controls (n = 3). * $p < 0.05$; ** $p < 0.01$.

Supplementary Fig. 2 Cell viability and chemosensitivity of Huh-7-trans, Huh-7-DN and HLE cells exposed to chemotherapeutic agents with/without GANT61. Cell viability of Huh-7-trans (**A**), Huh-7-DN (**B**) and HLE (**C**) cells exposed to GANT61. Chemosensitivity of Huh-7-trans, Huh-7-DN and HLE cells exposed to doxorubicin or cisplatin with/without GANT61 5 μM for 24 hours determined by MTT assay (**D-I**). n = 3, * $p < 0.05$; ** $p < 0.01$.

Supplementary Fig. 1

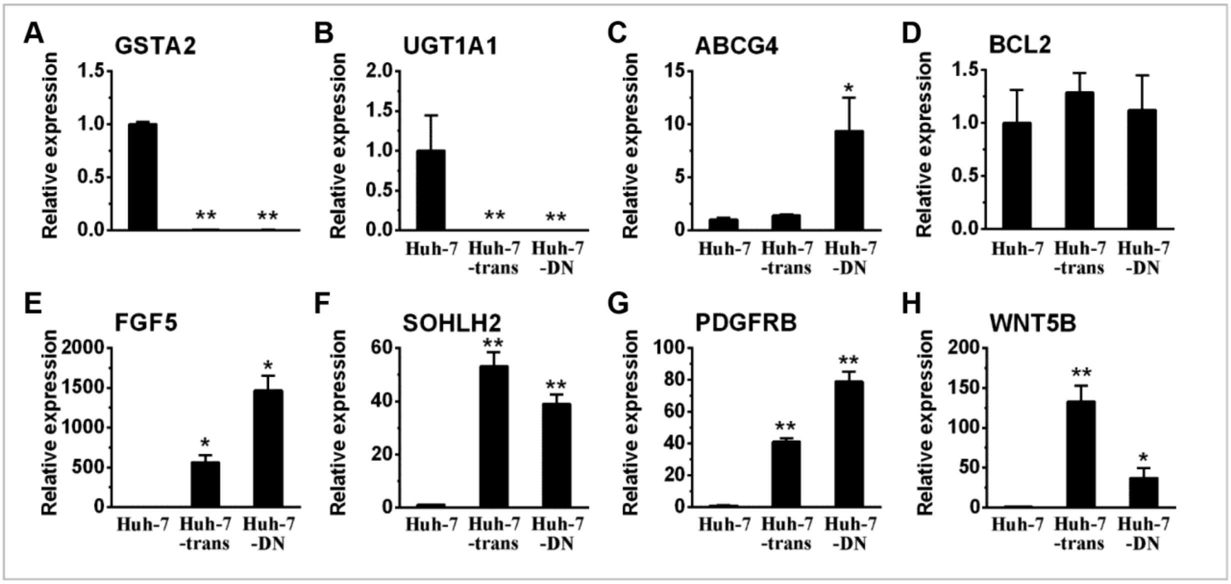

Supplementary Fig. 2

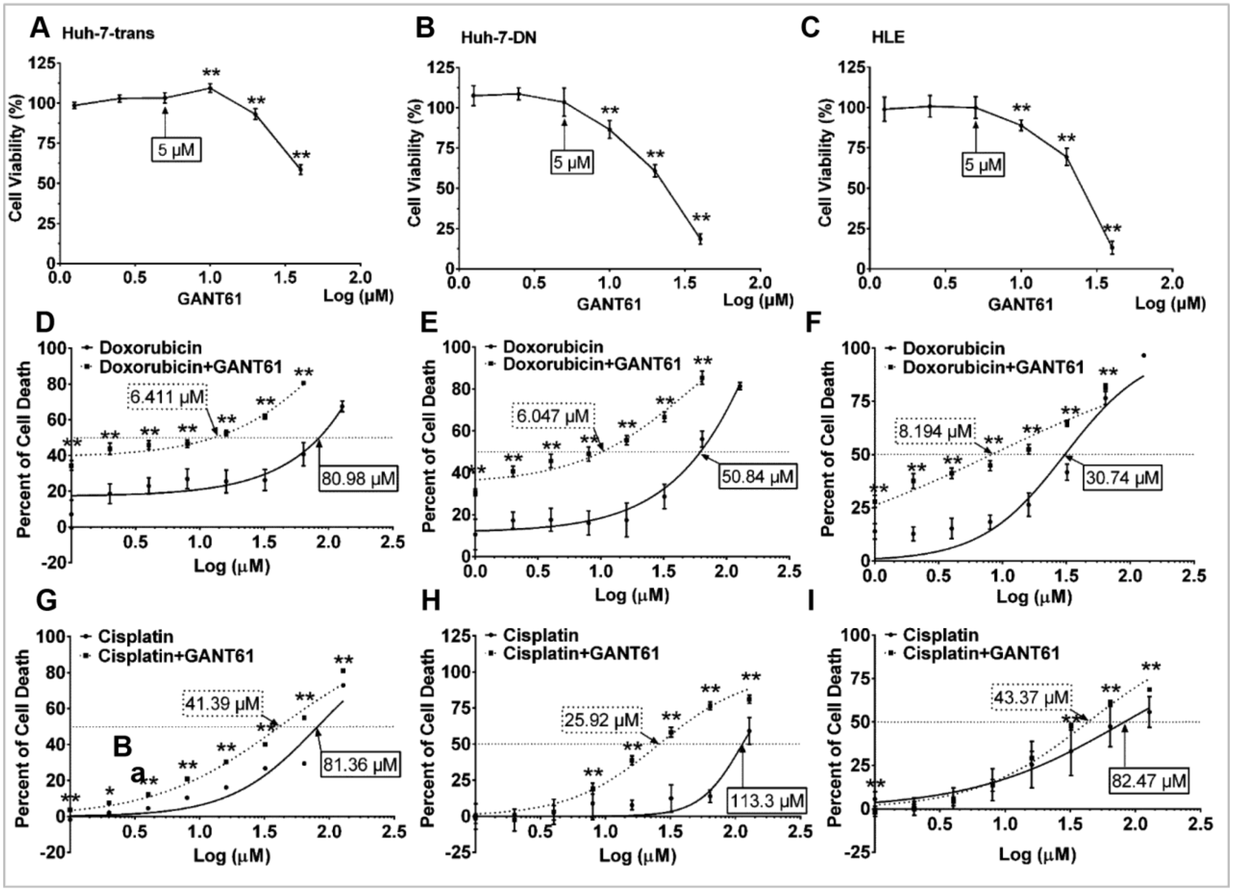

Supplement: Supplementary file 1 [file JCMM-24-4298-s001.pdf]
